# Spectrums of Opportunistic Infections and Malignancies in HIV-Infected Patients in Tertiary Care Hospital, China

**DOI:** 10.1371/journal.pone.0075915

**Published:** 2013-10-25

**Authors:** Jiang Xiao, Guiju Gao, Yanmei Li, Wen Zhang, Yunfei Tian, Yinxiu Huang, Wenjing Su, Ning Han, Di Yang, Hongxin Zhao

**Affiliations:** Center for Infectious Diseases, Ditan Hospital, Capital Medical University, Beijing, the People’s Republic of China; Fundacion Huesped, Argentina

## Abstract

**Background:**

HIV-related opportunistic infections (OIs) and malignancies continued to cause morbidity and mortality in Chinese HIV-infected individuals. The objective for this study is to elucidate the prevalence and spectrums of OIs and malignancies in HIV-infected patients in the Beijing Ditan Hospital.

**Methods:**

The evaluation of the prevalence and spectrums of OIs and malignancies was conducted by using the clinical data of 834 HIV-infected patients admitted in the Beijing Ditan hospital from January 1, 2009, to November 30, 2012.

**Results:**

The prevalence and spectrums of OIs and malignancies varied contingent on geographic region, transmission routes, and CD4 levels. We found that tuberculosis was most common OI and prevalence was 32.5%, followed by candidiasis(29.3%), *Pneumocystis pneumonia*(PCP)(22.4%), cytomegalovirus(CMV) infection(21.7%), other fungal infections(16.2%), mycobacterium avium complex(MAC)(11.3%), cryptococcosis(8.0%), progressive multifocal leukoencephalopathy(PML)(4.4%), Cerebral Toxoplasmosis(3.5%) and *Penicillium marneffei* infection(1.4%); while Lymphoma(2.9%), Kaposi’s sarcoma(0.8%) and cervix carcinoma(0.3%) were emerged as common AIDS-defining malignancies. Pulmonary OI infections were the most prevalent morbidity and mortality in patients in the AIDS stage including pulmonary tuberculosis (26.6%) and PCP (22.4%). CMV infection(21.7%) was most common viral infection; Fungal OIs were one of most prevalent morbidity in patients in the AIDS stage, including oral candidiasis (29.3%), other fungal infection (16.2%), Cryptococcosis (8.0%) and *Penicillium marneffei* infection (1.4%). We found the low prevalence of AIDS-defining illnesses in central neural system in this study, including progressive multifocal leukoencephalopathy (4.4%), cerebral toxoplasmosis (3.5%), tuberculosis meningitis (3.2%), cryptococcal meningitis (2.4%) and CMV encephalitis (1.1%). In-hospital mortality rate was 4.3 per 100 person-years due to severe OIs, malignancies, and medical cost constraints.

**Conclusions:**

The prevalence and spectrums of OIs, malignancies and co-infections were discussed in this study. It would help increase the awareness for physicians to make a diagnosis and empirical treatment sooner and plan good management strategies, especially in resource limited regions.

## Introduction

By the end of 2011, 740,000 people were estimated to be living with HIV/AIDS in China[1], HIV led to immunosuppression that allowed OIs and malignancies to cause diseases in HIV-infected patients. OIs led to frequent morbidity and mortality among HIV-infected individuals, and it mostly depended on CD4 levels, types of transmission route and geographic regions. 

Despite the availability of National Antiretroviral Treatment Programs (NFATP) in China[2,3], HIV-related clinical diseases (including OIs and malignancies) continued to cause morbidity and mortality in Chinese HIV-infected individuals. Some HIV-infected patients were not aware of HIV infection until OIs became the first indicator of their disease; some patients were aware of HIV infection but did not administer antiretroviral(ART) regimens due to skepticism[4] or some social factors; and some HIV-infected patients were taking ART medications but experiencing virological and immunological failure due to poor adherence. HIV-related OIs and malignancies increased morbidity and mortality, which shortened the lifespan of HIV-infected population and increased social-economic burdens on China.

The clinico-epidemiological spectrums of OIs and malignancies in HIV-infected population in China were rarely reported. Therefore it was important to promote awareness of physicians to make a right diagnosis and empirical treatment sooner, in particular, in resource limiting regions. The Beijing Ditan Hospital is a tertiary care hospital in China, which provide high-quality care and treatment to HIV/AIDS patients. The objective for this study is to elucidate prevalence and spectrums of OIs and malignancies in 834 HIV-infected patients in Ditan Hospital.

## Methods

This analysis was approved by the institutional review board (IRB) of Ditan Hospital, the Capital Medical University, Beijing. Per the IRB review, individual informed consent was waived because this analysis used the currently existing data collected during the course of routine treatment and care. The data were reported in aggregate.

### Patients selection

This retrospective observational study was carried out at the Center for Infectious Diseases, Beijing Ditan Hospital, the largest tertiary care hospital for HIV/AIDS patients in North China. The covering area of hospital included most provinces in North China, Northeastern and Northwestern china. The hospital also catered to some patients from provinces in South and Southwestern China. 

**Figure 1:**
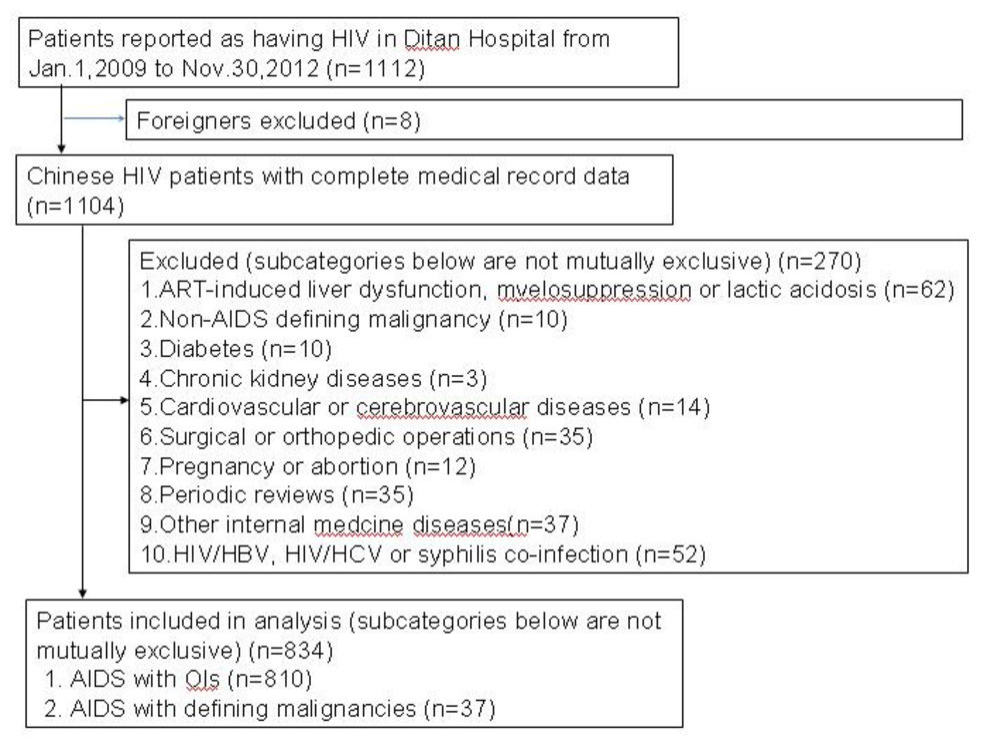
Study flow diagram.

We reviewed a series of 1112 HIV-infected patients who were admitted to Beijing Ditan Hospital between January1, 2009 and November30, 2012. We excluded some overseas’ patients, those diagnosed with non-AIDS-defining illnesses and malignancies, those who co-infected HBV, HCV or syphilis without other OIs or malignancies, the ones who had abortions, pregnant patients, and patients with some internal diseases or who had surgeries(Figure 1). The admitted patients presented some symptoms presumed to be due to OIs and malignancies, who needed evaluation and treatment of HIV-related diseases. Patients completed a face-to-face, paper-and-pencil questionnaire eliciting data on age, gender, ethnicity, marital status, transmission routes and address.

### Diagnosis of OIs, malignancies or co-infections

The diagnosis of OIs and malignancies was taken in accordance with the guideline recommended by the United States Center for Disease Control and Prevention(CDC)[5]. Laboratory tests were performed for blood routine test, liver and kidney functions, serum lipid panels and fasting plasma glucose. The CD4 cell count and percentage were evaluated using FACS calibur flow cytometer. Blood, urine, stool, sputum, bronochoalveolar lavage fluid(BALF) and even cerebrospinal fluid(CSF) were collected and used for culture and identification of species of pathogen depending on patients’ symptoms and signs. Fine needle aspiration(FNA) for pathological demonstration was performed in lymphadenopathy and mucocutaneous mass for evaluating aetiology.

Pulmonary tuberculosis was confirmed by the isolation, culture and identification of *mycobacterium tuberculosis*, or positive acid-fast bacilli(AFB) in sputum or BALF samples. Nucleic acid amplification test was applied to blood or sputum samples, and γ-IFN release assay(IGRA) was applied to blood samples to evaluate the patients with clinical presentations who were suspected HIV-related tuberculosis. For patients with symptoms and signs of extrapulmonary tuberculosis, pathological demonstration and AFB in samples of fine needle aspiration of lymph nodes and pleural fluid were required to be performed. The tuberculosis meningitis was diagnosed based on compatible systemic symptoms and signs, and cerebrospinal fluid(CSF) analysis indicated pleocytosis with mononuclear predominance, elevated protein and low glucose, and exclusion of cryptococcal meningitis. 

The etiologic diagnosis of tuberculosis in HIV-infected patients was difficult, and some patients with negative samples underwent antibiotic treatment for 2 weeks so that bacterial infection could be cured, patients still having fever, cough and night sweat would underwent anti-tuberculosis treatment, tuberculosis was empirically diagnosed due to clinical improvement after empirical anti-tuberculosis treatment.

Oropharyngeal candidiasis were diagnosed based on its features with painless, creamy white, plaque-like lesions in tongue surface or oropharyngeal mucosa which can be scraped off with tongue depressor.

*Pneumocystis pneumonia*(PCP) was confirmed by compatible clinical symptoms such as subacute onset of progressive dyspnea, fever, nonproductive cough and hypoxemia. Computerized tomography (CT) scan demonstrated patchy ground-glass attenuation. Induced sputum and BALF samples were collected to stain the cyst wall with Gomori methenamine silver. The etiologic diagnosis of PCP in HIV-infected patients sometimes was difficult, and some patients with compatible clinical symptoms and CT results but without positive BALF samples would underwent anti-PCP treatment, PCP was empirically diagnosed due to clinical improvement after empirical anti-PCP treatment.

Cytomegalovirus(CMV) viremia was detected based on CMV PCR and pp65 antigen assay. CMV retinitis was diagnosed according to recognition of characteristic retinal changes during an ophthalmoscopic examination. Endoscopic examination combined with histopathologic identification of intranuclear or intracytoplasmic inclusions was useful tool for diagnosis of CMV colitis. CMV neurologic disease was diagnosed based on compatible neural symtoms and positive CMV-DNA or pp65 antigen assay in CSF. 

Detection of serum cryptococcal antigen was used to initially screen cryptococcosis, and patients with positive serum cryptococcal antigen, and with symptoms and signs suggestive of cryptococcal meningitis, should have an lumbar puncture with CSF examination, india ink or CSF cryptococcal antigen assay. 

Penicilliosis was confirmed based on isolation and identification of *penicilliosis marneffei* from blood culture or by histopathologic demonstration of organisms in FNA samples. 

The diagnosis of other fungal infection was based on compatible clinical symptoms and measurement of serum (1,3)-ß-D-glucan[6], a cell wall component of most pathogenic fungi, and exclusion of PCP[6]. The culture, isolation and identification of species of fungi in HIV-infected patients were difficult, and some patients with compatible clinical symptoms, positive results of serum (1,3)-ß-D-glucan and exclusion of PCP would underwent anti-fungal treatment, and other fungal infection was empirically diagnosed due to clinical improvement.

Mycobacterium avium complex(MAC) disease was diagnosed based on isolation, culture and identification of MAC from culture of blood, BALF, focal infection sites and bone marrow. MAC infection should be suspected in patients with positive AFB but negative IGRA results who had poor anti-tuberculosis treatment.

Due to lack of brain biopsy, Toxoplasmic encephalitis was empirically diagnosed by clinical symptoms and focal neurological abnormalities, identification of one or more mass lesions by CT scan or magnetic resonance imaging (MRI) of the brain, and seropositive anti-toxoplasma IgG. 

AIDS-defining malignancies, including Kaposi’s sarcoma, lymphoma and cervix carcinoma were diagnosed based on their characteristic pathological demonstration. 

### Statistical analysis

The data were analyzed using SPSS 16.0(SPSS Institute, Chicago IL, USA). The demographic information included age, gender, ethnicity, marital status, transmission routes and address. Descriptive analysis was carried out to evaluate the percentage, mean and standard deviation(SD). The prevalence of OIs and malignancies based on geographic distribution, transmission routes, different CD4 levels and abnormalities in laboratory finding were expressed in percentages. The data were presented as median with interquartile range(IQR) when statistical distribution was skewed. χ^2^ test was applied in the prevalence of different OIs between the above and below 200cells/ul group and *p* value<0.05 was considered statistically significant.

## Results

### Demographic profile

278 patients were excluded from the sample of 1112 patients admitted in Beijing Ditan Hospital from January1, 2009 to November30, 2012. In total, 810 had OIs(72.8%), and 37 had AIDS-defining malignancies(3.3%) (See Figure 1).

Of 834 clinical records analyzed (Table 1), 695(82.8%) were male and 139(17.2%) were female patients, and the mean age of the study population was 39.2±11.4 years. The patients came from different provinces in China, 46.6% of patients were from North China, followed by 21.5%, 17.4%, 10.3% and 4.2% of the patients from Southern, Northeastern, Southwestern and Northwestern China. The most common transmission route was homosexual contact (48.7%), followed by extra-marital heterosexual contact (24.6%), transfusion(21.3%), drug addiction(4.1%) and vertical transmission(1.3%). CD4 cell counts were calculated in all study population, 494 patients (59%) had CD4 counts less than 50cells/ul, followed by 18.0%, 14.4% and 8.6% of the patients with 50-100, 100-200 and more than 200cells/ul, respectively.

**Table 1:** Demographic Characteristics and Prevalence of OIs and Malignancies.

**Characteristics**	**Total (%)**	**Male (%)**	**Female (%)**
Total patients	834(100)	695(82.8)	139(17.2)
Age(yrs)	39.2±11.4	39.1±11.4	39.4±11.6
**Geographic distribution (%)**			
Northeast China	145(17.4)	134(16.1)	11(1.3)
Northwest China	35(4.2)	32(3.9)	3(0.3)
North China	389(46.6)	329(39.4)	60(7.2)
South China	179(21.5)	139(16.7)	40(4.8)
Southwest China	86(10.3)	61(7.2)	25(3.1)
**Transmission-risk categories (%)**			
Homosexuality	406(48.7)	375(45.0)	31(3.7)
Heterosexuality	205(24.6)	170(20.4)	35(4.2)
Drug addiction	34(4.1)	29(3.9)	5(0.2)
Transfusion	178(21.3)	113(13.9)	65(7.4)
Vertical transmission	11(1.3)	8(1.0)	3(0.3)
**CD4 distribution (%**)			
CD4≤50 cells/ul	492(59.0)	403(48.3)	89(11.7)
50<CD4≤100 cells/ul	150(18.0)	131(15.7)	19(2.3)
100<CD4≤200 cells/ul	120(14.4)	100(12.0)	20(2.2)
CD4>200 cells/ul	72(8.6)	61(7.3)	11(1.3)
**AIDS-associated clinical diseases (%**)			
Tuberculosis	271 (32.5)	227 (27.2)	44 (5.3)
Candidiasis	245 (29.3)	202 (24.2)	43 (5.1)
PCP	187 (22.4)	163 (19.5)	24 (2.9)
CMV infection	181 (21.7)	148 (17.7)	33 (4.0)
Other fungal infection	135 (16.2)	112 (13.4)	23 (2.8)
MAC	94 (11.3)	85 (10.3)	9 (1.0)
Cryptococcosis	67 (8.0)	53 (6.3)	14 (1.7)
PML	37 (4.4)	30 (3.6)	7 (0.8)
Cerebral Toxoplasmosis	29 (3.5)	23 (2.7)	6 (0.8)
Lymphoma	27 (2.9)	21 (2.2)	6 (0.6)
*Penicillium Marneffei*	12 (1.4)	12 (1.4)	0 (0.0)
Kaposi's Sarcoma	7 (0.8)	6 (0.6)	1 (0.1)
Cervix carcinoma	3 (0.3)	--	3 (0.3)

PCP:pneumocystis pneumonia; CMV: cytomegalovirus; MAC: *Mycobacterium avium* complex; PML: progressive multifocal leukoencephalopathy.

Routine blood test results indicated that anaemia was found in 66.9% of the study population, and neutropenia was present in 39.2% of these patients. Liver dysfunction was found in 30.4% of the patients. Serum lipid profile indicated that a number of patients had hyperlipidemia and, 90.3% of which were with presented with lowered HDL, followed by 35.6% with increased TG level (Table 2).

**Table 2:** Laboratory results in 834 patients with HIV/AIDS.

**Variable**	**Range**	**Mean ± SD**	**Abnormality (%)**
Neutrophil count (×10^9^/L)	2.00-8.00	4.00±3.11	39.2
Neutrophil (%)	50.00-75.00	68.4±17.0	54.4
Lymphocyte count (×10^9^/L)	1.00-5.00	0.93±0.94	66.0
Haemoglobin (g/L)	120.0-160.0	108.8±22.9	66.9
PLT count (×10^9^/L)	100.0-300.0	208.8±101.6	28.9
CD4 cell count (cells/ul)	706-1125	76.4±101.1	99.8
ALT (U/L)	0.0-40.0	41.6±60.3	30.4
AST (U/L)	0.0-40.0	42.5±56.5	30.3
T-BIL (umol/L)	0.0-18.8	10.6±20.1	6.8
ALB (g/L)	35.0-53.0	33.4±6.9	62.1
BUN (mmol/L)	1.70-8.30	4.5±3.2	10.2
Cr (umol/L)	59.00-104.00	62.9±28.6	54.8
TG (mmol/L)	0.57-1.71	1.6±1.1	35.6
TC (mmol/L)	2.90-5.68	3.5±1.1	33.4
LDL (mmol/L)	0.00-3.36	2.1±0.8	6.8
HDL (mmol/L)	1.09-1.92	0.7±0.4	90.3
FPG(mmol/L)	4.16-6.44	5.6±1.9	28.3

PLT: Platelet; ALT: Alanine aminotransferase; AST: Aspartate aminotransferase ; T-BIL: Total Bilirubin; ALB: Albumin; BUN: Blood urea nitrogen; Cr: Serum creatinine; TG: triglyceride ; TC: Total cholesterol; LDL: Low-density lipoprotein; HDL: high-density lipoprotein; FPG: Fasting plasma glucose.

### Spectrum of OIs, malignancies and co-infections

**Figure 2:**
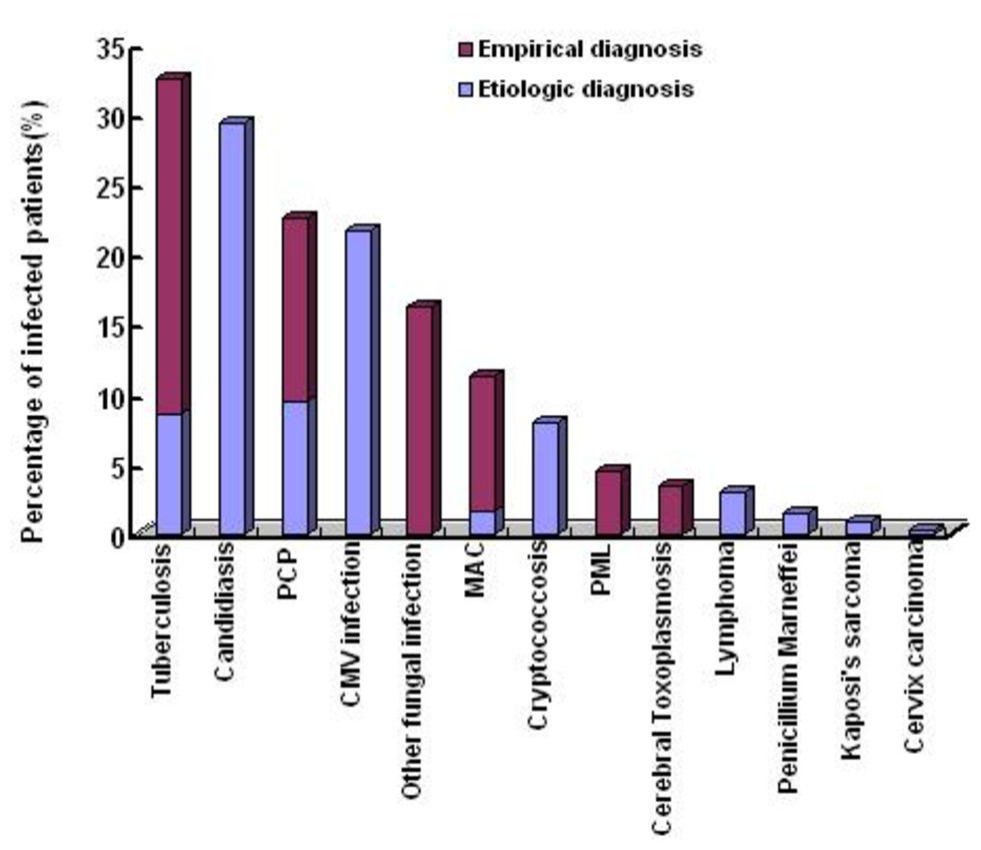
Spectrum of OIs and Malignancies in HIV-infected Patients based on etiologic and empirical diagnosis in the Beijing Ditan Hospital from January 1, 2009 to November 30, 2012. **Note**: PCP:pneumocystis pneumonia; CMV: cytomegalovirus; MAC: *Mycobacterium avium* complex; PML: progressive multifocal leukoencephalopathy.

Tuberculosis was most common OI among 834 clinical records analyzed(Table 1), and the prevalence rate was 32.5%. This was followed by candidiasis(29.3%), PCP(22.4%), CMV infection(21.7%), other fungal infections(16.2%), MAC(11.3%), cryptococcosis(8.0%), PML(4.4%), Cerebral Toxoplasmosis(3.5%) and *Penicillium Marneffei* infection(1.4%); Lymphoma(2.9%), Kaposi’s sarcoma(0.8%) and cervix carcinoma(0.3%) were emerged as common AIDS-defining malignancies(See Figure 2).

**Figure 3:**
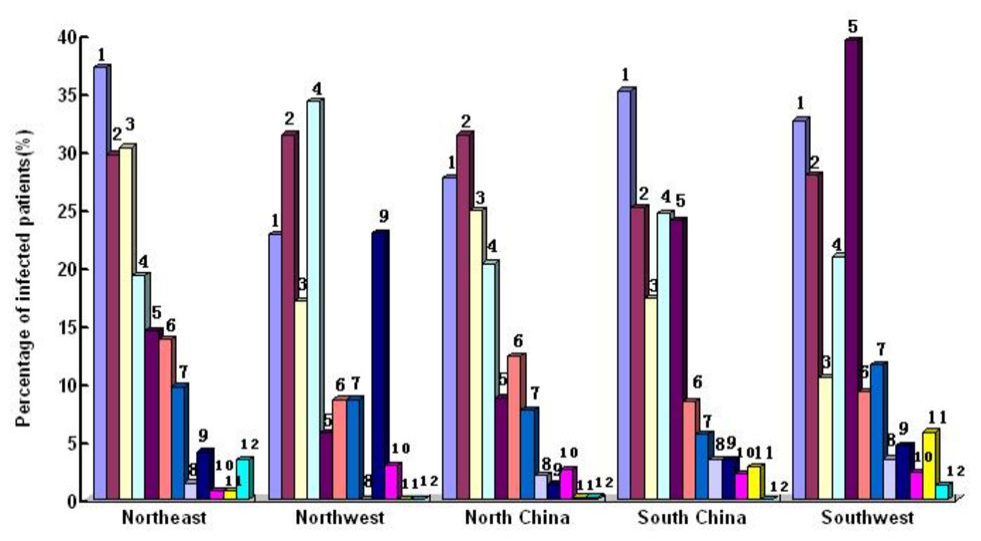
Prevalence of OIs and Malignancies Related to Geographic Regions in the Study Subjects. 1.Tuberculosis; 2.Candidiasis; 3.PCP; 4.CMV infection; 5.Other fungal infection; 6.MAC; 7.Cryptococcosis; 8. PML; 9. Cerebral Toxoplasmosis; 10. Lymphoma; 11.Penicillium Marneffei infection; 12. Kaposi's Sarcoma.

**Figure 4:**
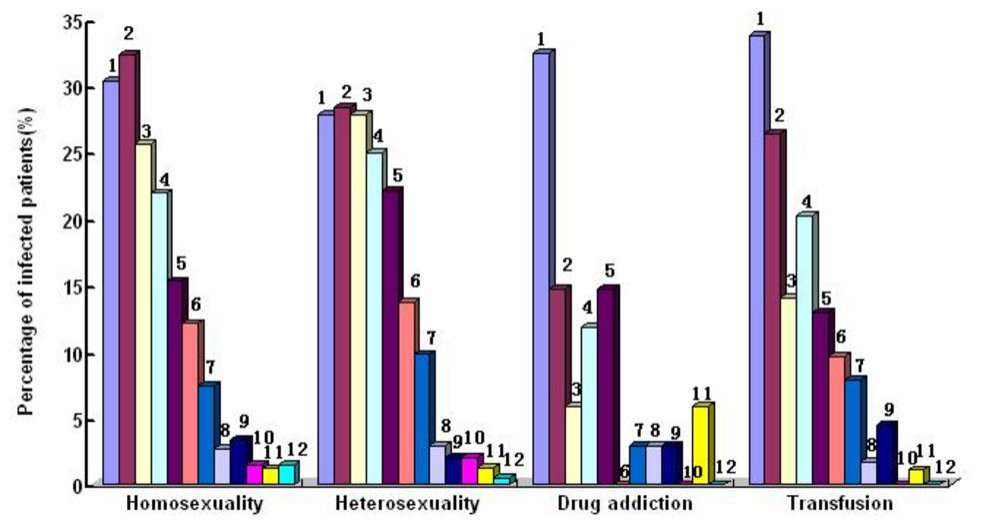
The Prevalence of OIs and Malignancies Related to Transmission Routes in the Study Subjects. 1.Tuberculosis; 2.Candidiasis; 3.PCP; 4.CMV infection; 5.Other fungal infection; 6.MAC; 7.Cryptococcosis; 8. PML; 9. Cerebral Toxoplasmosis; 10. Lymphoma; 11.Penicillium Marneffei infection; 12. Kaposi's Sarcoma.

**Figure 5:**
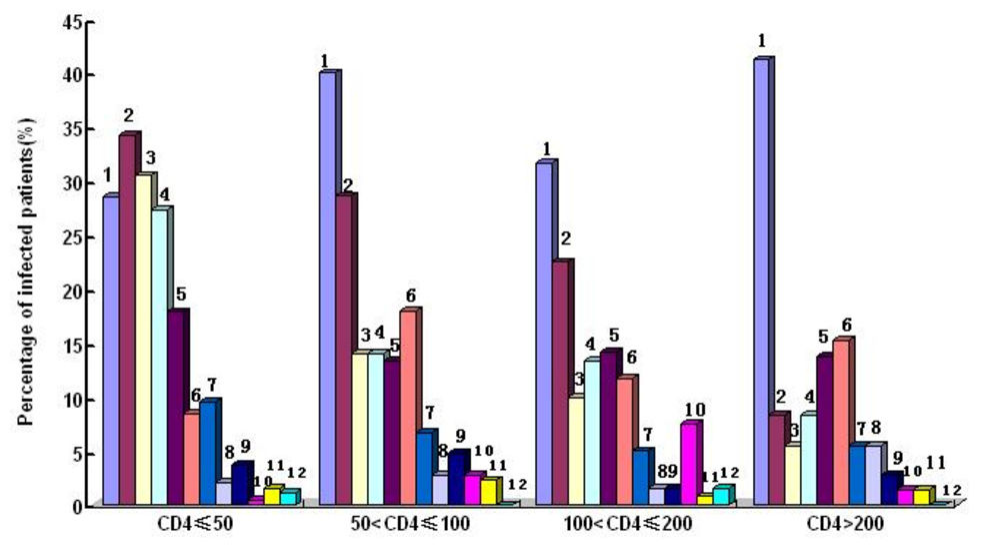
The Prevalence of OIs and Malignancies related to CD4 counts in study subjects. 1.Tuberculosis; 2.Candidiasis; 3.PCP; 4.CMV infection; 5.Other fungal infection; 6.MAC; 7.Cryptococcosis; 8. PML; 9. Cerebral Toxoplasmosis; 10. Lymphoma; 11.Penicillium Marneffei infection; 12. Kaposi's Sarcoma.

The percentages of different OIs and malignancies based on etiologic and empirical diagnosis were shown in Figure 2. The distributions of different OIs and malignancies in terms of geographic regions, transmission routes and CD4 levels were shown in Figure 3, 4 and 5. The prevalence and spectrums of OIs varied across different regions in China, fungal infections including *penicillium Marneffei* infection mainly occurred in the Southern and Southwestern China; not a single case of *penicillium Marneffei* infection was found in patients from the Northwestern China, while cerebral toxoplasmosis was more common in Northwestern China than in other part of China. The distribution of different OIs and malignancies also varied regarding different transmission routes. CMV infection, Kaposi’s sarcoma, and lymphoma were found mainly in the patients with sexual transmission routes, especially in homosexual contact. Comparatively, various OIs exhibited a higher prevalence in patients with CD4 fewer than 200cells/ul (p<0.05). 

Pulmonary OIs were the most prevalent morbidity and mortality cause in patients in the AIDS stage. Among these, tuberculosis(32.5%) were the most common OIs in respiratory system, in which 8.5% were etiologically diagnosed based on positive samples, while 24% were empirically diagnosed based on compatible symptoms and negative samples. PCP(22.4%) were the second most common OIs in respiratory system(9.4% etiologically diagnosis and 13.0% empirically diagnosis) (See Figure 2), and PCP was most common cause of death in this study(See Table 3).

**Table 3:** AIDS-related OIs and Malignancies and In-hospital Deaths in 834 Patients.

**Diagnosis**	**Prevalence (%)**	**Median CD4 counts (IQR)**	**In-hospital deaths n(%**)^1^
Tuberculosis			
Pulmonary tuberculosis	222 (26.6)	74.5(1-312)	4(2.8)
Lymphoid tuberculosis	74 (8.9)	67.5(1-276)	--
Tuberculous pleuritis	52 (6.2)	89.7(3-351)	--
Tuberculous meningitis	27 (3.2)	57.4(3-210)	8(5.6)
Candidiasis	245 (29.3)	45.3(1-211)	--
PCP	187 (22.4)	35.8(1-211)	47(33.1)
CMV infection			
CMV retinitis	34 (4.2)	63.5(1-219)	--
CMV cephalitis	9 (1.1)	14.2(1-68)	3(2.1)
CMV colitis	1(0.1)	5	1(0.7)
CMV viremia	137 (16.4)	43.3(1-230)	--
Other fungal infection	135 (16.2)	57.6(1-278)	--
MAC	94 (11.3)	77.3(1-278)	--
Cryptococcosis			
Cryptococcal antigenemia	67(8.0)	50.0(2-289)	--
Cryptococcal meningitis	20(2.4)	49.9(2-218)	9(6.3)
PML	37 (4.4)	128.6(18-246)	2(1.4)
Cerebral Toxoplasmosis	29 (3.5)	55.1(1-210)	3(2.1)
Lymphoma			
Diffuse large B cell lymphoma	12 (1.4)	105.8(3-208)	3(2.1)
Bunkitt’s lymphoma	6 (0.7)	126.8(12-212)	3(2.1)
Unclassified lymphoma	9(1.1)	91.6(5-178)	9(6.3)
*Penicillium Marneffei*	12 (1.4)	49.1(4-210)	1(0.7)
Kaposi's Sarcoma	7 (0.8)	53.6(10-157)	--
Cervix carcinoma	3 (0.3)	403(187-611)	1(0.7)

^1^means causes of death in this table was confirmed in 94 patients, while another 48 patients were dead caused by unexplained infections or malignancies and intracranial space-occupying lesion.

PCP:pneumocystis pneumonia; CMV: cytomegalovirus; MAC: *Mycobacterium avium* complex; PML: progressive multifocal leukoencephalopathy.

Tuberculosis is a systemic disease and it was the most common OI in this study. In total, 26.2% of these patients were diagnosed with pulmonary tuberculosis while 17.3% of the patients had extrapulmonary tuberculosis that included lymphoid tuberculosis(8.9%), pleuritis(6.2%) and meningitis(3.2%), in which 11.8% of patients had pulmonary and extrapulmonary tuberculosis. Pulmonary and meningitis tuberculosis were common causes of death in HIV/tuberculosis co-infection (Table 3).

Viral infection was another prevalent morbidity and mortality cause in patients in the AIDS stage that mainly included CMV infections (Table 3). CMV infection was the most common viral infection and the prevalence rate was 21.7%. 16.4% of these patients with median CD4 counts 43.3cells/ul presented with CMV viremia without any end-organ diseases, while 4.2% presented with CMV retinitis and 1.1% with encephalitis with median CD4 counts 63.5 and 14.2cells/ul, respectively. A patient with CD4 count 5 cells/ul was diagnosed with CMV colitis based on endoscopic examination combined with histopathologic demonstration that indicated that CMV-related end-organ diseases often occurred in patients with CD4 counts less than 100cells/ul. This will increase the awareness of physicians to establish diagnosis and perform treatment of CMV-related end-organ diseases.

Fungal OIs were one of most prevalent morbidity in patients in the AIDS stage that included oral candidiasis(29.3%), other fungal infection(16.2%), Cryptococcosis(8.0%) and *Penicillium Marneffei* infection(1.4%)(Figure 2 and Table 1).Fungal infection including *Penicillium Marneffei* infection often emerged in patients living in Southern or southwestern China.

In this study, a few AIDS-defining malignancies were found(Figure 2 and Table 1), viz. diffused large B cell lymphoma(1.4%), Bukitt’s lymphoma(0.7%), unclassified lymphoma(1.1%), Kaposi’s sarcoma(0.8%) and cervix carcinoma(0.3%), which were diagnosed based on pathological demonstration.

**Figure 6:**
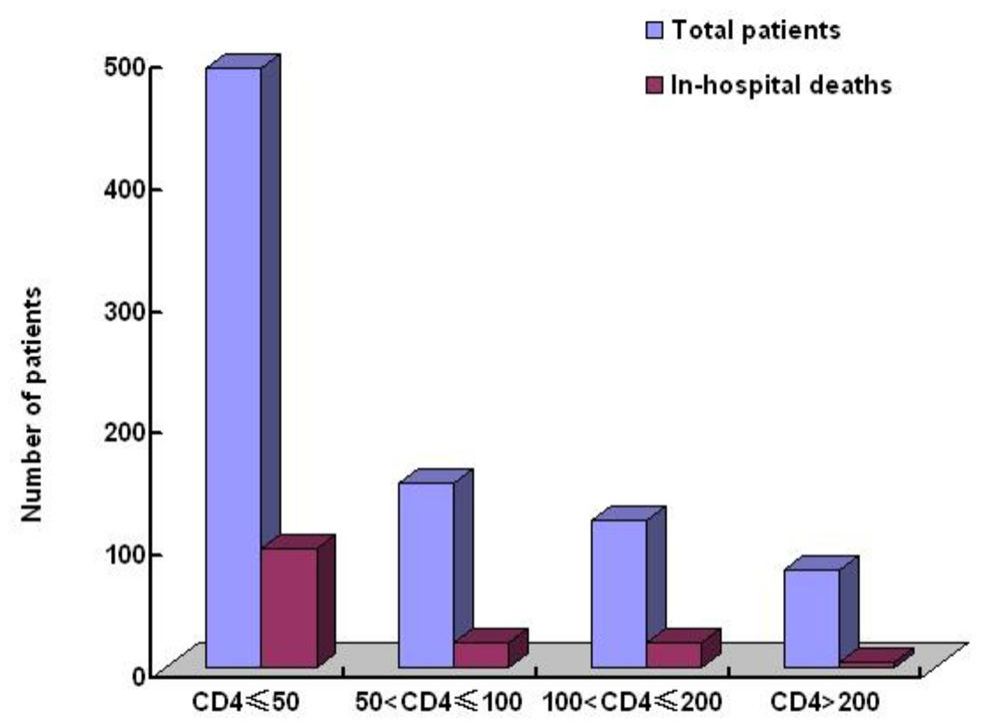
Distribution of CD4+T Counts and In-hospital deaths in the Study Subjects.

142 patients(4.3 per 100 person-years) died in this study(Figure 6 and Table 3), including 48 in-hospital deaths caused by unexplained infection, malignancies or intracranial space-occupying lesions. In another 94 deaths, most of them were due to PCP (47 patients), central neural system infection (22 patients), AIDS-defining malignancies (16 patients). 117 patients who died in hospital had CD4 counts less than 100cells/ul. 

## Discussion

In China, HIV-infected patients was treated based on NFATP, which was administered through a low-technology, community-based treatment model[3,4], while OIs and malignancies were treated in HIV/AIDS centers of tertiary care hospital due to poor expertise and facilities in community level hospital. The Beijing Ditan Hospital is the largest center for HIV/AIDS diagnosis and treatment and the cover area included all provinces in China. In this retrospective observational study, 834 HIV-infected patients came from different geographic regions in China and hence the spectrum of OIs and malignancies in current study may apply to different regions in China.

An accurate evaluation of the prevalence and spectrum of OIs and malignancies helps to plan good management strategies. It was reported that[7], in a series of 135 hospitalized HIV-infected patients from north India, the five most common AIDS-defining illnesses were tuberculosis(71%), candidiasis(39.3%), PCP(7.4%), cryptococcal mentingitis(3.7%) and toxoplasmosis(3.7%). Another retrospective study in 89 cases in Lebanon with OIs reported[8] that the most common OIs were cerebral toxoplasmosis(21%) and fungal infection(17%), which indicated that the prevalence and spectrum of OIs in HIV-infected population varied contingent on geographic locations and social-economic conditions. In this study, we found that most common OIs were tuberculosis, candidiasis, PCP, CMV infection, other fungal infection, MAC and Cryptococcosis.

In this study, the majority of the patients (54.9%) were in the age group of 20-40 years, and men were predominantly affected, which was consistent with the findings in Indian[9] and Euro-American[10] studies. Thus OIs and malignancies occurred in economically productive years in these HIV-infected individuals that increased social-economic burden in China.

 OIs occurred in HIV-infected patients were contingent on geographic locations, and the frequencies and types of OIs varied from region to region due to different climates and social-economic conditions. In this study, HIV-infected patients came from different regions in China, and the findings revealed that other fungal infection[11] and *Penicillium Marneffei* infection[12] were more common in Southern and Southwestern China, where the ecological conditions and humid climate were similar to that of Southeast Asia. But these fungal infections were rarely found in the northwestern China due to dry climate. We also found that HIV-infected patients in Northwestern China showed higher prevalence of Cerebral Toxoplasmosis than that found in other parts of China. North-Western China is live-stock breeding area and therefore, HIV-infected patients were more prone to have Protozoan *toxoplasma gondiin* infection through contacting with animals. 

The most common risk of HIV transmission in this study was homosexual contact, found in 46.9% of patients, and this was followed by non-marital heterosexual contact (25.4%), which indicated that sexual contacts had become the most common transmission route of HIV infection in China. The findings by transmission routes revealed that OIs and malignancies such as CMV infection, lymphoma and Kaposi’s sarcoma were more common in sexual contacts. It was reported [13] that CMV infection and Kaposi’s sarcoma occurred in patients who had contacted with men who have sex with men(MSM), and intravenous users that was similar to that of the findings in this study.

The findings in this study revealed that prevalence of transfusion transmission was 21.3%. It was reported [14] that former plasma donors across central China sold plasma to unscrupulous collectors in unsanitary conditions during the early-mid-1990s, resulting in untold numbers of HIV infection. Chinese government had banned paid blood donation since 2000 and free antiretroviral therapy in NFATP was provided to former plasma donors to control HIV transmission. Patients who had blood transfusion during the early-mid-1990s were not aware of HIV infection until OIs or malignancies became the first indicator of their disease. In this study, 21.3% of patients had blood transfusion during the early-mid-1990s, got OIs or malignancies after a decade of latent period. 

The culture, isolation and identification of species of pathogens such as *mycobacterium tuberculosis, Pneumocyst*, some *invasive fungi* and *Mycobacterium avium complex* in some HIV-infected patients were difficult, and due to lack of brain biopsy, pathological diagnosis of Toxoplasmic encephalitis and PML was also difficult, empiric diagnosis and treatment was taken in patients with compatible clinical and radiographic results, which helped us minimize the false negative diagnosis.

The important finding in this study revealed that pulmonary OI infections were the most prevalent morbidity cause in patients in the AIDS stage, and tuberculosis and PCP were most common OIs in respiratory system, which was similar with results in Indian [15]. The process of diagnosis of pulmonary OI infections was well planned, and HIV-infected patients with symptoms in respiratory tract underwent *mycobacterium tuberculosis* culture in induced sputum and BALF samples, pathological demonstration and PCR detection for *PCP* or *mycobacterium tuberculosis* in BALF samples, IGRA assay[16] for *mycobacterium tuberculosis* and thin-section computerized tomography scan. Empiric diagnosis and anti-tuberculosis treatment was taken in some patients with negative samples who were suspected of being infected with tuberculosis.

The prevalence of mycobacterium tuberculosis infection was 32.5% in this study, which was similar to reports in Indian studies [17]. Tuberculosis infection was a systemic disease, and with increasing degrees of immunodeficiency, extrapulmonary tuberculosis was more common. In this study, we found that pulmonary tuberculosis was the most widespread (26.6%) followed by lymphadenitis (8.9%), pleuritis (6.2%) and meningitis (3.2%), in which 11.8% of patients had pulmonary and extrapulmonary tuberculosis. This indicated that that tuberculosis infection was one of most widespread OIs in Chinese HIV-infected patients. We also found that tuberculosis infection was prevalent at any levels of CD4 counts. These findings indicated that appropriate tuberculosis management strategies should be planned and implemented in Chinese HIV-infected population.

PCP was found in 22.4% of patients in this study, which was diagnosed based on etiology and empirical treatment. *Pneumocystis jirovecii* cannot be cultivated routinely, cytopathologic demonstration of this organism in BALF was required for etiologic diagnosis. Empirical diagnosis was based on compatible clinical symptoms and CT results, negative cytopathologic demonstration of organism and clinical improvement after empirical anti-PCP treatment. we found that prevalence of etiologic and empirical diagnosis were 9.4% and 13.0%, respectively. serum (1,3)-ß-D-glucan may be elevated in patients with PCP, but the assay ’s sensitivity and specificity for establishing a PCP diagnosis were problematic [18]. In this study, we did not adopt elevated serum (1,3)-ß-D-glucan as diagnostic standard for PCP. 

The findings in this study revealed that CMV infection was the most common viral infection. Most of CMV infection, especially CMV related end-organ diseases, occurred in HIV-infected patients with immune suppression, typically in those with CD4 count less than 50cells/ul [19]. In this study, 21.7% of the patients were found to have CMV infection, in which 4.2% and 1.1% had found retinitis and cephalitis, and the median CD4 counts were 63.5cells/ul and 14.2cells/ul, respectively. Endoscopic examination combined with biopsy and histopathologic examination in HIV-infected patients with digest tract symptoms was performed and one case of CMV colitis had been found. 

The occurrence of oral candidiasis was recognized as an indicator of immune suppression, and those were often found in HIV-infected patients with CD4 counts fewer than 200cells/ul. The prevalence of oral candidiasis in this study was 29.3% with the median CD4 counts 45.3cells/ul, which indicated that the occurrence of oral candidiasis helped physicians in resource constrained regions in China to diagnose the clinical progression of HIV infection. 

The findings in this study revealed that prevalence of *Penicillium Marneffei* infection was 1.4%. Penicilliosis was known to be endemic in Southeast Asia and southern China, and the definitive diagnosis of penicilliosis was based on culture, isolation and identification of species of *Penicillium Marneffei* from blood samples or other clinical specimens. We found that penicilliosis was mainly diagnosed in patients from southern or southwestern China, and most cases of penicilliosis were observed in patients who had CD4 cell counts less than 100cells/ul(mean CD4 cell counts was 49.1cells/ul), This will increase the awareness of physicians to establish diagnosis and perform treatment of penicilliosis in patients with less than 100cells/ul from southern or southwestern China.

Except *Candidiasis, Penicillium Marneffei, Cryptococcosis*, The culture, isolation and identification of species of other fungi in HIV-infected patients were difficult, and some patients with compatible clinical symptoms, positive results of serum (1,3)-ß-D-glucan and exclusion of PCP was empirically diagnosed as other fungal infection, and the prevalence was 16.2% in this study. Other fungal infection was diagnosed with (1,3)-ß-D-Glucan assay with standard reference value defining >100pg/ml, which should be differentiated with PCP. It was reported [6] that (1,3)-ß-D-Glucan was correlated with HIV-related PCP. In this study, PCP was diagnosed based on characteristic clinical presentation, thin-section computerized tomography scan and histopathological demonstration *pneumocystis jirovecii* in BALF, but not on elevated serum (1,3)-ß-D-glucan while other fungal infection was diagnosed based on systemic symptoms and (1,3)-ß-D-Glucan >100pg/ml.

We found the low prevalence of AIDS-defining illnesses in central neural system in this study, including PML (4.4%), cerebral toxoplasmosis (3.5%), tuberculosis meningitis (3.2%), cryptococcal meningitis (2.4%) and CMV encephalitis (1.1%), which contributed to increased mortality in HIV-infected patients. Lubar puncture and MRI scan were required in HIV-infected patients with central nervous system (CNS) manifestation and different pathogen detections were performed in cerebrospinal fluid to differentiate various OI infections. We found that etiologic diagnosis of intracranial space-occupying lesions were most difficult in this study. Due to high risk and poor adherence, we hardly took brain biopsy in these patients. space-occupying lesions were empirically diagnosed based on clinical symptoms, focal neurological abnormalities and identification by CT scan or magnetic resonance imaging(MRI) of the brain, which may be the reason for low prevalence of OIs and malignancies in central neural system in this study.

We also found some AIDS-defining malignancies including lymphoma, Kaposi’s saroma and cervix carcinoma, which were definitively diagnosed based on pathological demonstration. Fine needle aspiration combined with pathological demonstration was performed in lymphadenopathy and mucocutaneous mass, which helped physicians to differentiate malignancies with OI infections. We found that incidence ratio of AIDS-related Kaposi’s saroma and cervix carcinoma in this study was similar with that reported in Euro-American[20,21], while prevalence of lymphoma was lower than that in EUROSIDA cohort[22], which indicated that pathogenicity of lymphoma should be further understood in HIV-infected patients. 

In-hospital mortality in this study was 4.3 per 100 person-years, and 94 of these deaths were due to severe OIs and malignancies while 48 were caused by unexplained infections or malignancies and intracranial space-occupying lesions due to cost constraints and unaffordable further diagnosis and treatment. 

Our study has some additional limitations. First, the study is not a meta-cohort observational analysis in this cohort, with the increase in the number of patients in the observational cohort, the results achieved in this study should remain to be further validated. The second limitation is inherent biases based on observational data (i.e. bias such as referral of complex patients from other centers, or patients dying before arriving the hospital might increase or reduce the frequency of admissions) and without control group for comparison.

In conclusion, the prevalence and spectrums of OIs and malignancies varied contingent on geographic region, transmission route and CD4 level. Tuberculosis, candidiasis, PCP, CMV infection, other fungal infection and MAC emerged as the most common OIs. In-hospital mortality rate was considerable due to severe OIs, malignancies and cost constraints. It would help to increase the awareness of physicians to come up with right diagnoses and implement empirical treatment sooner and plan good management strategies, especially in resource constrained regions in China.
