# Correction: Spectrums of Opportunistic Infections and Malignancies in HIV-Infected Patients in Tertiary Care Hospital, China

**DOI:** 10.1371/annotation/bbbcd86d-200e-49d6-a3e3-aef3e083fab2

**Published:** 2014-01-03

**Authors:** Jiang Xiao, Guiju Gao, Yanmei Li, Wen Zhang, Yunfei Tian, Yingxiu Huang, Wenjing Su, Ning Han, Di Yang, Hongxin Zhao

The sixth author's name was spelled incorrectly. The correct name is: Yingxiu Huang 

